# Immune-mediated mechanisms and maternal-fetal interface dysfunction in obstetric antiphospholipid syndrome

**DOI:** 10.3389/fimmu.2025.1722080

**Published:** 2025-11-26

**Authors:** Guangyu Ma, Jinbiao Han, Rui Gao, Lang Qin

**Affiliations:** 1 Reproductive Medical Center, Department of Obstetrics and Gynecology, West China Second University Hospital, Sichuan University, Chengdu, China; 2 Key Laboratory of Birth Defects and Related Diseases of Women and Children, Ministry of Education, West China Second University Hospital, Sichuan University, Chengdu, China; 3 Department of Obstetrics and Gynecology, West China Second University Hospital, Sichuan University, Chengdu, China; 4 Meishan City Maternal and Child Health Hospital, Meishan, China

**Keywords:** obstetric antiphospholipid syndrome, antiphospholipid antibodies, maternal-fetal interface, trophoblast dysfunction, immune dysregulation

## Abstract

Obstetric antiphospholipid syndrome (OAPS) is a complex autoimmune disorder that significantly compromises pregnancy, manifesting as recurrent miscarriage, stillbirth, placental insufficiency, and preeclampsia. Its increasing prevalence underscores the pressing need to elucidate its multifaceted pathogenic mechanisms to improve maternal and fetal outcomes. While traditionally attributed to thrombosis driven by antiphospholipid antibodies (aPL), emerging evidence indicates that OAPS can disrupt placental perfusion, impair trophoblast proliferation and invasion, and compromise placental angiogenesis even in the absence of overt thrombotic events. Beyond direct effects on trophoblasts and vascular remodeling, aPLs profoundly perturb the immune milieu at the maternal–fetal interface, encompassing complement activation, excessive formation of neutrophil extracellular traps (NETs), dysfunction of decidual natural killer cells and macrophages, and dysregulated B cell responses. These immune-mediated alterations collectively establish a sustained pro-inflammatory environment that undermines placental development and predisposes to adverse pregnancy outcomes. This review provides a comprehensive synthesis of the immunopathogenic mechanisms of OAPS that extend beyond thrombosis, and emphasizes the intricate crosstalk between immune cells and the complement-NET axis. A deeper understanding of these immune-mediated pathways may inform the development of targeted therapeutic strategies to optimize maternal and fetal outcomes in affected pregnancies.

## 1 Introduction

**Figure 1:**
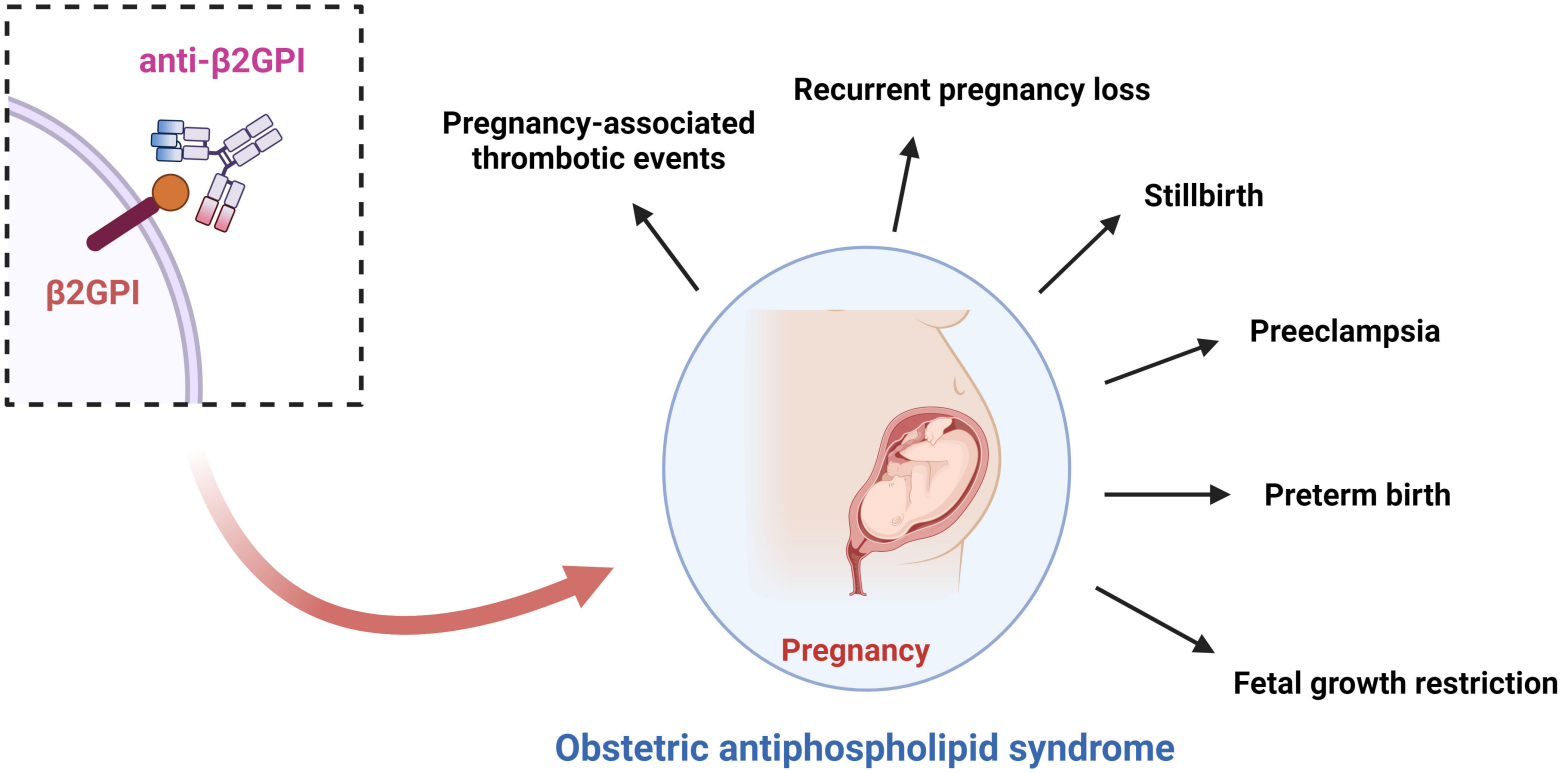
Schematic representation of pregnancy complications associated with OAPS. This figure was created with Biorender.com.

Antiphospholipid syndrome (APS) is an acquired systemic autoimmune disorder mediated by the persistent presence of antiphospholipid antibodies (aPLs). Clinically, it is characterized by arterial and/or venous thrombosis and pregnancy-related complications (1–3). The commonly used assays for detecting aPLs include lupus anticoagulant (LA), anticardiolipin antibodies (aCL), and anti-β2-glycoprotein I antibodies (anti-β2GPI) (1, 4). In the general population, the prevalence of aPLs among healthy women of reproductive age is approximately 1%-5%, whereas it rises to 10%-29% in women with adverse pregnancy outcomes, suggesting that aPLs are important contributors to pregnancy complications (2, 5, 6). Obstetric APS (OAPS) represents a distinct subtype of APS, typically manifesting as recurrent miscarriage, fetal death, placental dysfunction, and preeclampsia, and it poses substantial risks to both maternal and fetal outcomes (1, 7) (**Figure 1**).

Current research on OAPS has predominantly focused on the thrombotic mechanisms mediated by aPLs, which are believed to contribute to placental vascular occlusion and pregnancy loss (8). Combined anticoagulation therapy with low-molecular-weight heparin and low-dose aspirin can improve pregnancy outcomes to some extent; however, approximately 30% of patients remain refractory to conventional anticoagulation (9–11), indicating that the underlying pathogenesis has not been fully elucidated. Although early studies proposed that microthrombosis at the maternal–fetal interface represented the primary pathogenic event in OAPS, subsequent histopathological analyses revealed that overt placental thrombosis is relatively uncommon in these patients (12). Moreover, some patients develop pregnancy complications even in the absence of apparent thrombosis, suggesting that additional immune-mediated or inflammatory mechanisms may contribute to OAPS pathogenesis beyond thrombosis.

Beyond coagulation abnormalities, recent studies have demonstrated that aPLs can directly impair trophoblast function, disrupt placental angiogenesis, and disturb immune homeostasis at the maternal-fetal interface, thereby fostering a pro-inflammatory milieu that is detrimental to pregnancy maintenance. This review aims to provide a comprehensive overview of the immunopathogenic mechanisms of OAPS that extend beyond thrombosis and to explore emerging immunotherapeutic targets, with the ultimate goal of offering new insights to optimize clinical management and improve maternal-fetal outcomes.

## 2 Placental physiologies: the foundation for understanding OAPS pathogenesis

A clear understanding of placental physiology is fundamental to elucidating how OAPS leads to pregnancy complications. Placental function primarily depends on trophoblast differentiation and activity, the integrity of the villous architecture, and the remodeling of maternal blood flow.

The human placenta consists of an extensive network of finger-like projections known as villi, most of which float within the intervillous space filled with maternal blood (13). These floating villi are covered by the multinucleated syncytiotrophoblast, a terminally differentiated epithelial layer that is continuously renewed by the underlying villous cytotrophoblasts, which provide proliferative and fusogenic support (14). Villous cytotrophoblasts proliferate and fuse to form the syncytiotrophoblast in a process termed syncytialization. Approximately 3–4 weeks after syncytialization, syncytiotrophoblast nuclei aggregate, undergo chromatin condensation, and experience DNA fragmentation as part of physiological apoptosis, forming characteristic syncytial knots (15, 16). These knots, along with small syncytiotrophoblast fragments shed into the maternal circulation, collectively constitute trophoblast debris (17, 18). Beneath the trophoblastic layers lies the villous stromal core, which contains fetal blood vessels, fibroblasts, and macrophages.

In a subset of villi, certain villous cytotrophoblasts differentiate into extravillous trophoblasts (EVTs), which penetrate the syncytiotrophoblast and invade the maternal decidua, anchoring the placenta to the uterine wall (19). EVTs also infiltrate uterine spiral arteries-initially narrowing the vascular lumen to modulate flow, and later remodeling them into large-caliber, low-resistance vessels devoid of vasoconstrictive capacity. This transformation ensures optimal maternal perfusion to sustain the increasing metabolic demands of the growing placenta and fetus.

Decidual immune cells, including macrophages and natural killer (NK) cells, play pivotal roles in the establishment and maintenance of pregnancy as well as in placental development. Through delicate crosstalk with fetal-derived trophoblasts, they mediate maternal-fetal immune tolerance and promote trophoblast differentiation and development (20–22). However, this finely tuned immune balance is highly vulnerable to disruption.

In the context of OAPS, aPLs can disturb the local immune microenvironment and directly impair trophoblast function. *In vitro* studies have demonstrated that aPLs decrease trophoblast viability and hinder both syncytialization and invasive capacity (23, 24). Impaired syncytialization compromises not only maternal-fetal exchange but also placental endocrine function, leading to placental insufficiency. Meanwhile, restricted EVT invasion disrupts spiral artery remodeling, providing the pathological basis for preeclampsia. Placental insufficiency and preeclampsia represent typical placenta-mediated complications of OAPS (1), underscoring the central role of trophoblast injury in its pathogenesis.

## 3 Classical pathogenic mechanisms: placental microthrombosis and ischemic injury

### 3.1 Placental microthrombosis and ischemic injury

Placental microthrombosis is considered a classical pathological feature of OAPS-related pregnancy complications. Clinical studies have proposed the well-known “two-hit” model: First, antiphospholipid aPL induce a procoagulant state by activating endothelial cells and platelets, as well as impairing vascular function, representing the initial first hit; second, in the presence of additional prothrombotic stimuli or tissue injury, inflammatory and coagulation pathways are further amplified, with endothelial cells upregulating anti-β2 glycoprotein I (β2GPI), which constitutes the second hit and ultimately drives thrombus formation (25). By binding to β2GPI, aPL can activate endothelial cells, platelets, and the complement system, thereby promoting microthrombosis within the decidua and intervillous space (25). In addition, certain aPL directly target prothrombin or β2GPI-prothrombin complexes, enhancing the conversion of prothrombin to thrombin and thereby amplifying the coagulation cascade. These microthrombi restrict placental perfusion and cause local ischemia-hypoxia, leading to trophoblast necrosis, fibrosis, and disorganization of the intervillous structure (26). Recurrent or sustained vascular occlusion may further impair placental vascular autoregulation and disturb maternal-fetal exchange, providing the pathological basis for abnormal embryonic development and pregnancy loss.

### 3.2 Annexin V protective shield disruption

In addition to the “two-hit” model, disruption of the Annexin V protective shield is recognized as another classical pathogenic pathway of OAPS. The externalized phosphatidylserine on the surface of syncytiotrophoblasts can trigger contact-dependent coagulation. Under normal pregnancy conditions, Annexin V binds to these sites and forms a lattice-like protective layer that effectively prevents excessive coagulation (27–31). *In vitro* studies have demonstrated that aPL can displace Annexin V from the surface of placental explants or syncytialized trophoblasts, thereby disrupting this anticoagulant shield, enhancing the binding of cells to prothrombin, and accelerating plasma coagulation reactions, ultimately conferring a marked prothrombotic tendency (32–36). The resulting hypercoagulability promotes fibrin deposition around villi and thrombosis within the intervillous space, leading to obliteration of the intervillous channels, trophoblast ischemia, necrosis, and loss of function (37).

### 3.3 Limitations of the thrombosis-based mechanistic study

Nevertheless, clinical and pathological studies have shown that thrombosis alone cannot fully explain all OAPS-related pregnancy complications. A systematic pathological review of 580 placentas revealed that intravascular or intervillous thrombi were not commonly observed in placentas from aPL-positive women (12). Furthermore, while anticoagulation therapy provides some protective benefit against early pregnancy loss, its efficacy is limited in late pregnancy loss and other complications (38). Importantly, placental pathology more frequently demonstrates inflammation, necrosis, and immune cell infiltration rather than isolated vascular occlusion. These findings indicate that the pathogenesis of OAPS is not confined to thrombosis, and emerging evidence highlights the critical roles of direct immune-mediated placental injury and maternal-fetal interface immune dysregulation.

## 4 Immunopathogenic mechanisms in obstetric antiphospholipid syndrome

### 4.1 Complement system activation

#### 4.1.1 Evidence for complement activation in OAPS

Emerging evidence highlights complement dysregulation as a pivotal factor in OAPS development. APS patients with complicated pregnancies exhibit elevated circulating C5a and C5b-9 compared to healthy controls (39). In addition, placental analyses have shown enhanced deposition of C4d, C3b, and C5b-9 at the maternal-fetal interface, which correlates with intrauterine fetal loss (40, 41). Clinically, reduced maternal serum complement levels have been associated with adverse pregnancy outcomes, including fetal growth restriction, preterm birth, and preeclampsia (42, 43). Mechanistic studies support a causal role for complement: mice lacking C1q, C3, C4, C5, or factor B are protected from aPL-induced pregnancy complications, and pharmacological inhibition of complement similarly prevents these effects (39, 44–47). Collectively, these findings establish complement activation as a key driver of placental injury in OAPS and suggest it as a potential therapeutic target. Identifying the specific complement pathways involved is therefore critical for advancing targeted interventions.

#### 4.1.2 Complement activation: classical and alternative pathways in OAPS

**Figure 2:**
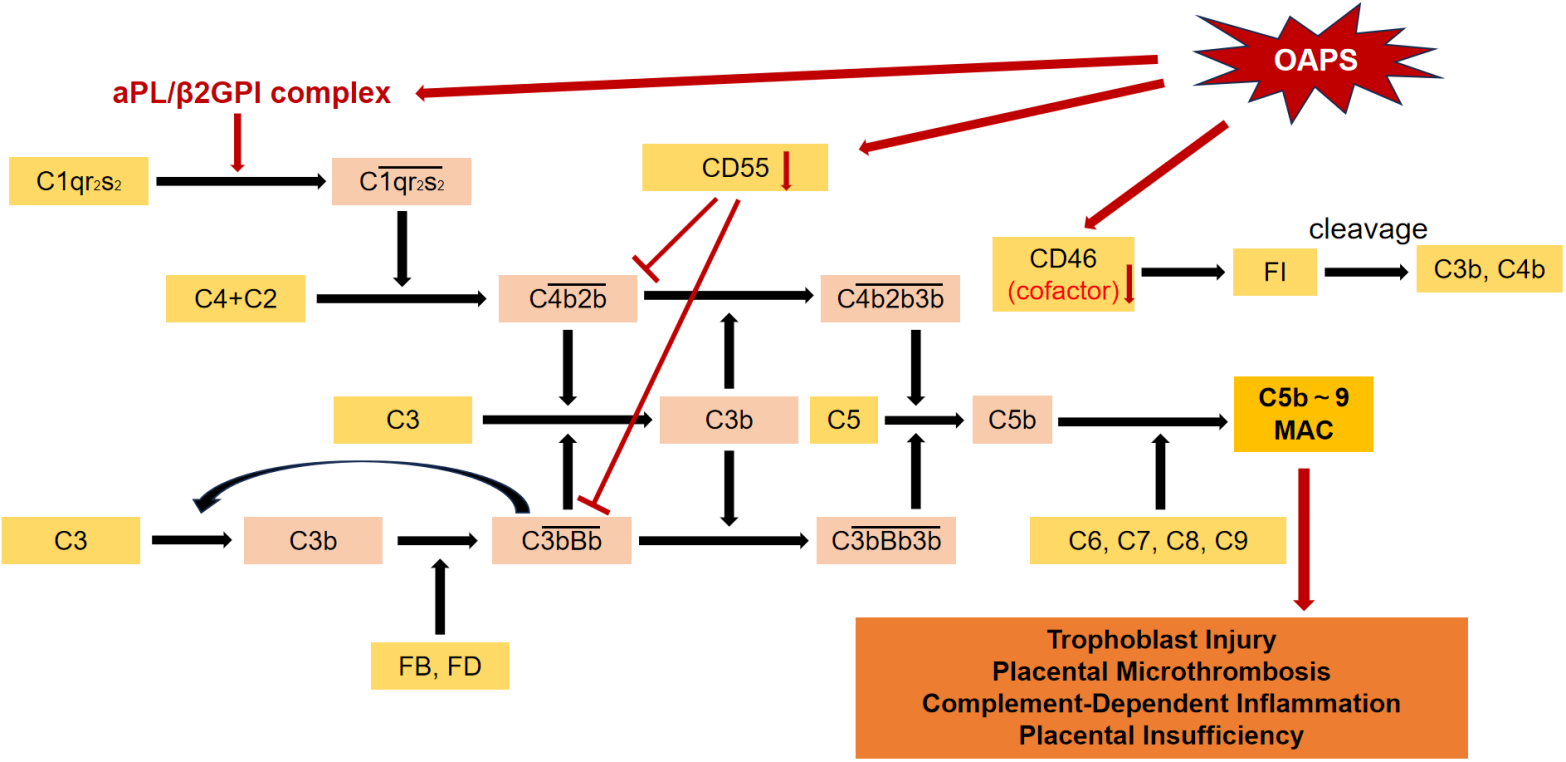
In OAPS, both the classical and alternative complement pathways are activated. Expression of complement regulators such as CD46 and CD55 is often reduced or dysregulated, which may impair physiological control of complement activity and thereby promote excessive cascade amplification.

In OAPS, complement activation is predominantly driven by the classical pathway, which is initiated when the C1 complex binds to antigen-antibody complexes **Figure 2**. This binding leads to the cleavage of C4 and C2, generating the C3 convertase (C4b2a). The C3 convertase then cleaves C3 into C3a and C3b, with C3b facilitating the assembly of the C5 convertase (C4b2a3b) (48). The cascade ultimately results in the formation of the membrane attack complex (MAC, C5b-9), which causes direct cell injury at the maternal-fetal interface and plays a central role in the pathogenesis of OAPS (49, 50). Placental C4d deposition correlates with adverse pregnancy outcomes, and C4 deficiency in mice reduces placental damage and fetal loss, highlighting its critical role in disease progression (40, 44).

Although both the classical and lectin pathways involve C4 cleavage, the lectin pathway has a limited role in OAPS. This is supported by the absence of mannose-binding lectin (MBL) in placental samples and by findings from double-knockout models deficient in both C1q and factor D (40). These observations emphasize that the classical pathway is the primary driver of complement activation in OAPS, making it a promising target for therapeutic intervention.

On the other hand, the alternative pathway amplifies complement activation in OAPS. This pathway is triggered by spontaneous C3 hydrolysis or direct recognition of activating surfaces, leading to the formation of C3(H_2_O). This molecule interacts with factors B and D to form C3 convertase (C3bBb), which is stabilized by properdin and enhances C3 cleavage through a positive feedback loop. The continued binding of C3b converts the C3 convertase into the C5 convertase, further promoting MAC formation (51). Elevated plasma Bb levels reflect sustained alternative pathway activity and correlate with disease severity (52). In animal models, C3 deficiency or its inhibition reduces fetal resorption and improves fetal growth (53). Similarly, factor B deficiency or treatment with factor B inhibitors in aPL-injected mice reduces fetal resorption, increases birth weight, and decreases C3 deposition in placental tissues (44, 46). These findings highlight the crucial role of the alternative pathway in OAPS progression.

Complement activation products, particularly C3a and C5a, intensify local inflammatory responses and promote coagulation. Activation of C5 enhances TNF−α expression, stimulates neutrophils to express tissue factor, and induces the release of anti-angiogenic molecules such as soluble fms-like tyrosine kinase-1 (sFlt−1), thereby disrupting angiogenesis and placental perfusion (54). These processes lead to placental hypoxia, increasing the risk of embryonic injury. In summary, the classical pathway initiates complement activation in OAPS, while the alternative pathway amplifies it through a pathogenic feed-forward loop. Together, these mechanisms drive persistent complement activity in affected pregnancies. Additionally, failure of local regulatory mechanisms contributes to complement-mediated placental injury.

#### 4.1.3 Dysregulation of negative regulation in complement activation in OAPS

Membrane-bound complement regulators, including membrane cofactor protein (MCP/CD46) and decay-accelerating factor (DAF/CD55), are essential for restraining complement activation and preserving immune tolerance at the maternal-fetal interface (55, 56). CD55 accelerates the decay of C3 and C5 convertases (C4b2a and C3bBb), limiting excessive C3 activation and preventing uncontrolled complement amplification. CD46 acts as a cofactor for Factor I, mediating the proteolytic inactivation of C3b and C4b into non-inflammatory fragments such as iC3b and C3dg, thereby reducing complement-mediated cytotoxicity and downstream inflammatory signaling (57–60).

In experimental models of obstetric APS, loss or impaired expression of these regulators increases placental complement deposition, promotes neutrophil infiltration, and elevates proinflammatory cytokine production, collectively impairing placental function. In DAF-deficient mice, this dysregulation leads to more severe placental inflammation and higher fetal resorption rates, highlighting the importance of local complement control in supporting fetal survival (61).

Human placental studies have reported variable expression of complement regulatory proteins in APS and OAPS. One study found similar CD46 and CD55 levels between APS patients and controls, but a consistent reduction in CD59 (39). In high-risk pregnancies, lower CD55 expression was associated with adverse outcomes, suggesting that impaired complement regulation may contribute to obstetric complications. However, a study reported elevated CD46 and CD55 expression in APS placentas, which may reflect a compensatory response to heightened inflammatory or oxidative stress rather than an intrinsic defect in complement control (62).

Together, these findings underscore the critical role of CD46 and CD55 in maintaining immune homeostasis at the maternal–fetal interface. Their context-dependent expression in OAPS, influenced by disease severity, gestational stage, and local inflammatory conditions, highlights the complex regulation of complement activation during pregnancy. Moreover, these observations support the potential of targeted complement modulation as a therapeutic strategy to improve obstetric outcomes.

### 4.2 Pathogenic mechanisms of OAPS mediated by NETs

#### 4.2.1 Structure and function of NETs

Neutrophil extracellular traps (NETs) are web-like structures released by neutrophils through a specialized form of programmed cell death known as NETosis. They are primarily composed of DNA, histones, and antimicrobial proteins such as myeloperoxidase (MPO), elastase, and cathepsin G (63). NETs play a crucial role in host defense by trapping and killing pathogens, thereby preventing their dissemination. However, under pathological conditions, excessive or dysregulated NET formation has been implicated in autoimmune disorders such as APS and OAPS, where NETs not only amplify inflammatory responses but also promote thrombosis and placental injury (64–67).

#### 4.2.2 Mechanisms of NETs formation in OAPS

**Figure 3:**
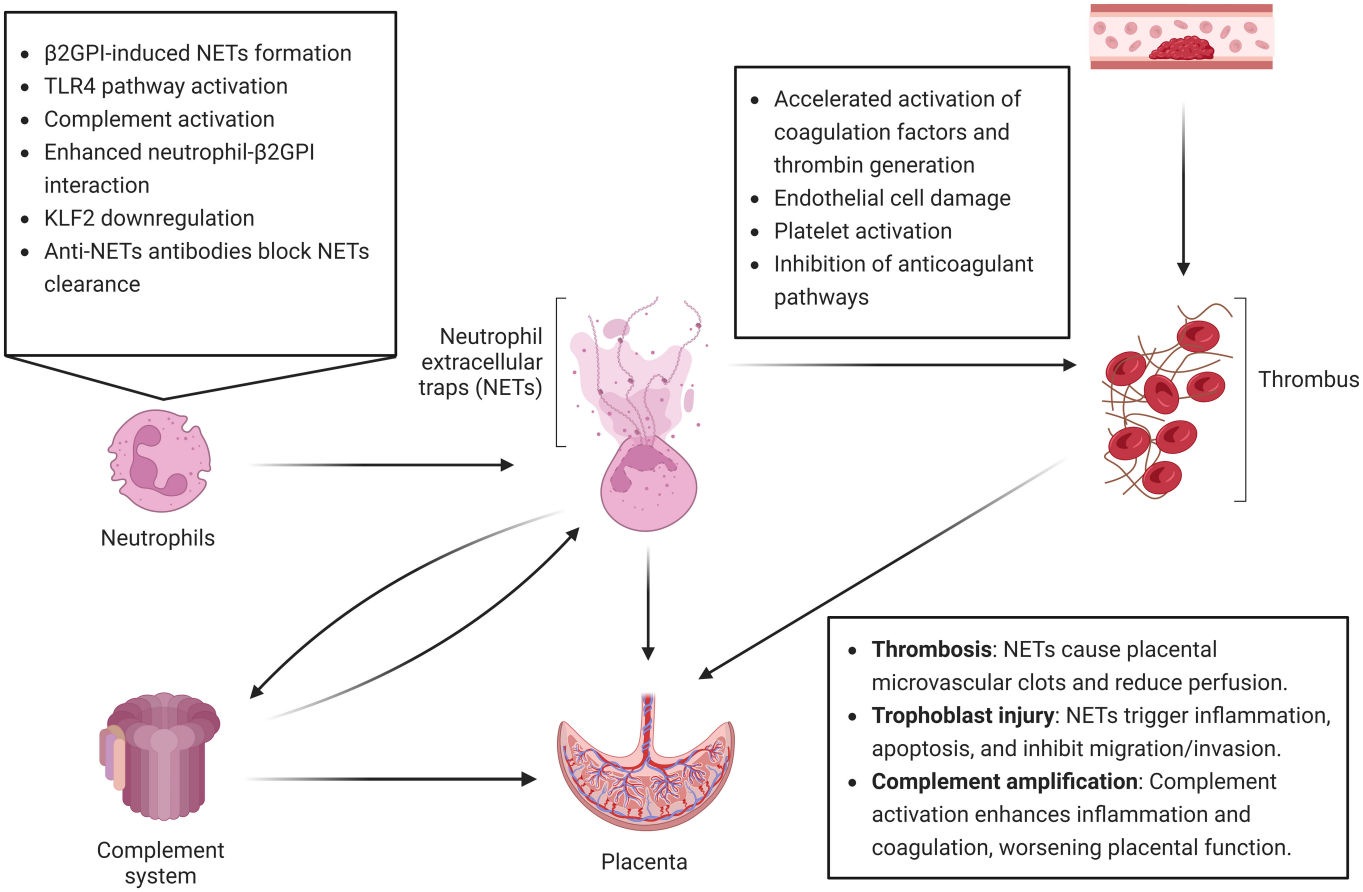
Schematic of NETs formation induced by OAPS and its contribution to placental injury. In OAPS, aPL bind to β2GPI on neutrophils and activate them through the TLR4 pathway, with complement activation and KLF2 downregulation further promoting NETs release. Crosstalk between NETs and complement amplifies inflammation and coagulation, while anti-NET antibodies impair NET clearance, prolonging their pathogenic effects. NETs contribute to placental injury through three major mechanisms: (1) promoting microvascular thrombosis and placental underperfusion; (2) inducing trophoblast inflammation and apoptosis while suppressing their migration and invasion, thereby impairing placental development; and (3) driving complement-mediated inflammatory–coagulant amplification loops, which exacerbate placental dysfunction and increase the risk of OAPS-related pregnancy complications. This figure was created with Biorender.com.

In OAPS, NETs contribute to disease pathogenesis through multiple mechanisms **Figure 3**. On one hand, they provide a scaffold that exposes negatively charged DNA and associated TF, thereby enhancing local coagulation and thrombin generation. On the other hand, NETs promote trophoblast inflammation and apoptosis while suppressing trophoblast migration and invasion, ultimately leading to placental dysfunction and adverse pregnancy outcome (66).

Clinical studies have demonstrated elevated circulating NET levels in patients with APS. Anti-β2GPI antibodies can bind β2GPI expressed on neutrophil surfaces, triggering NET release via reactive oxygen species (ROS) generation and Toll-like receptor 4 (TLR4)-dependent pathways (67). Moreover, neutrophils from OAPS patients show downregulation of the transcription factor Krüppel-like factor 2 (KLF2), which facilitates P-selectin glycoprotein ligand-1 (PSGL-1) clustering, enhanced adhesion to endothelial cells, increased NET formation, and augmented TF activity, all promoting a prothrombotic phenotype (68). *In vitro* studies further suggest that neutrophils from APS patients exhibit a lower threshold for spontaneous NETosis, indicating a primed state for NET release. aPLs, through interaction with β2GPI on neutrophil membranes, provide potent activation signals that amplify NET generation (67, 69). In addition, complement activation can indirectly strengthen neutrophil-β2GPI interactions, further enhancing NET release (70).

Furthermore, anti-NET antibodies have been detected in autoimmune diseases including APS, and may impair NET clearance, thereby prolonging their persistence in circulation. This persistence can lead to complement activation and amplify inflammatory and prothrombotic responses (71, 72).

#### 4.2.3 Mechanisms by which NETs promote thrombosis in APS

NETs are key mediators of thrombus formation in APS. Their lattice-like architecture not only physically ensnares platelets and erythrocytes but also exposes negatively charged DNA backbones capable of binding TF, thereby accelerating coagulation and thrombin generation. Following vascular injury, NET-associated TF interacts with factor VIIa, triggering activation of factors X and IX and initiating the extrinsic coagulation cascade (73, 74). Additionally, histones released from NETs can injure endothelial cells and activate platelets, amplifying local thrombogenicity (75). Proteases embedded in NETs can degrade tissue factor pathway inhibitor (TFPI), attenuating natural anticoagulant activity and promoting clot formation (76–78). NETs may also compromise other anticoagulant mechanisms, such as activated protein C (APC) function, leading to APC resistance. Elevated NET levels in APS patients have been correlated with impaired APC activity, potentially explaining their predisposition to recurrent venous and arterial thromboses (76, 79, 80).

#### 4.2.4 NETs-mediated placental injury in OAPS

In a murine model of obstetric APS, Girardi et al. (44) observed pronounced inflammation in the decidua accompanied by extensive neutrophil infiltration. Further studies demonstrated that depletion of neutrophils or inhibition of complement factor C5a significantly alleviated the pathological damage induced by antiphospholipid aPLs, indicating a central role of neutrophils and the complement system in the pathogenesis of obstetric APS (44). aPLs promote neutrophil recruitment through complement activation, exacerbating placental inflammation and inducing trophoblast apoptosis (81).

Moreover, aPL-induced NETs can activate BNIP3-mediated mitophagy, increasing reactive ROS production and further promoting trophoblast apoptosis, thereby aggravating placental injury and providing new molecular insights into APS pathogenesis (82). Mechanistic studies have shown that aPLs enhance NET formation in early-pregnancy APS patients by activating the AKT, ERK1/2, and p38 MAPK signaling pathways. These NETs not only impair trophoblast migration and invasion but also inhibit umbilical vein endothelial cell migration and angiogenesis, ultimately hindering placental development and leading to pathological pregnancies. In addition, dysregulation of extracellular matrix components, including matrix metalloproteinase-9 (MMP-9) and fetal fibronectin, may further compromise trophoblast function and exacerbate placental injury (83). Notably, treatment with DNase markedly alleviates these damages (66), suggesting that NETs are key mediators of placental injury in obstetric APS.

Currently, large-scale clinical studies evaluating the role of NETs in obstetric complications of APS and their association with disease onset, progression, and prognosis are lacking. Further investigation of NET-related mechanisms may provide novel targets for therapeutic intervention in obstetric APS.

#### 4.2.5 Crosstalk between NETs and complement in OAPS

A reciprocal amplification loop exists between NETs and the complement system, shaping the inflammatory and prothrombotic features of OAPS. Complement activation occurs not only on neutrophil surfaces but also on NETs after their release. Properdin, Factor B, and C3 have been shown to deposit on NETs (84–86). Moreover, NET-associated enzymes such as myeloperoxidase (MPO), cathepsin G, and proteinase 3 can modulate complement activity by binding soluble properdin or stabilizing properdin already attached to NETs (87). Experiments with isolated NETs in non-heat-inactivated serum reveal both consumption of complement components and assembly of the MAC on NET structures (86, 88).

Conversely, complement effectors also promote NET formation and prolong NET stability. Among them, C5a is a potent neutrophil activator that can initiate or amplify NETosis (89, 90). In the specific context of OAPS, β2-GPI may serve as a molecular bridge: it associates with NETs, increasing their immunogenicity, while anti-β2-GPI immune complexes activate the classical complement pathway, thereby reinforcing local inflammation and procoagulant signaling (91). This reciprocal interplay provides a mechanistic explanation of how antiphospholipid antibodies fuel both inflammation and thrombosis. It also highlights a therapeutic opportunity: targeting either NETs or complement could synergistically mitigate placental injury and adverse pregnancy outcomes in OAPS.

### 4.3 Immune cell interactions and dysregulation in OAPS

#### 4.3.1 B cell dysregulation and autoreactive antibody production

During normal pregnancy, B cells support humoral immunity and help maintain immune tolerance at the maternal-fetal interface. Regulatory B cell subsets produce anti-inflammatory cytokines such as IL-10 and IL-35, promoting Treg differentiation and restraining excessive Th1- and Th17-driven responses (92). In OAPS, regulatory B cell frequency and function are markedly reduced, compromising the tolerogenic microenvironment and facilitating aberrant immune activation, which contributes to autoantibody production and placental injury (93).

Breakdown of B cell tolerance is a central feature of OAPS. Serum levels of B cell-activating factor (BAFF), a key survival and differentiation factor for B cells, are elevated in early pregnancy among OAPS patients and correlate with adverse outcomes such as miscarriage (94). Elevated BAFF promotes the persistence of autoreactive B cells and the production of antiphospholipid aPLs, thereby driving inflammation, complement activation, and thrombosis (95). BAFF can also stimulate monocytes and other antigen-presenting cells to release pro-inflammatory cytokines including IL-6, IL-17, and IL-23 (96, 97), which further activate neutrophils and amplify inflammation. IgG-aPL can directly activate endothelial cells, inducing TF, IL-6, IL-8, and TNF-α expression, contributing to thrombosis and placental injury (98). Thus, BAFF serves both as a marker of inflammatory activity and as a key driver of OAPS immunopathology.

Autoreactive B cells in OAPS display abnormal activation pathways. They may be stimulated through TLR or BAFF signals independently of T cell help, generating short-lived plasmablasts that secrete low-affinity antibodies. Alternatively, they can undergo T cell-dependent follicular responses, differentiating into long-lived plasma cells that produce high-affinity antibodies (99, 100). In APS, B cells preferentially adopt T cell-dependent differentiation, accompanied by increased T follicular helper (Tfh) cells and decreased T follicular regulatory (Tfr) cells, highlighting the role of follicular maturation in sustaining aPL production (101). Antigen-presenting cells can also present intact β2GPI via HLA class II, further promoting helper T cell-mediated autoreactive responses (102).

Breakdown of B cell tolerance in OAPS is associated with several signaling abnormalities. Autoreactive B cells producing aPLs may derive from naturally polyreactive naïve B cells that bypass peripheral tolerance due to excessive survival cues. These cells can undergo aberrant germinal center reactions, lose their original polyreactivity, and develop strong autoreactive specificity (102). At the antibody level, IgG from OAPS patients shows altered glycosylation, including reduced fucosylation and sialylation, which enhance Fcγ receptor engagement and complement-mediated cytotoxicity. Distinct IgG subclasses also exhibit specific glycosylation patterns, further contributing to aPL pathogenicity (103, 104).

In summary, B cells drive OAPS pathogenesis through BAFF-mediated survival of autoreactive cells and aPL production, Tfh/Tfr imbalance that sustains autoreactive immune responses, and aberrant antibody glycosylation that increases the pathogenicity of aPLs.

#### 4.3.2 NK cell dysfunction and placental immune injury

In early pregnancy, the maternal-fetal interface is dominated by uterine NK cells, which account for roughly 70% of local lymphocytes. These cells, enriched around small arteries and glands, play essential roles in remodeling spiral arteries, supporting fetal development, and sustaining immune tolerance. Through interactions with HLA antigens on EVTs, such as HLA-G engaging inhibitory receptors KIR2DL4 and LILRB, NK cells receive targeted signals that prevent excessive immune responses (20, 105).

In the decidua of patients experiencing recurrent miscarriage, NK cell and macrophage dysfunction is common, characterized by increased NK cytotoxicity and heightened pro-inflammatory activity of macrophages (106–109). Elevated NK cell numbers during early pregnancy are correlated with pregnancy failure, whereas in normal gestation, NK cell counts gradually decline as pregnancy progresses (110). Placental bed biopsies from OAPS patients experiencing recurrent miscarriage show increased NK cell infiltration and heightened production of cytokines, such as IL-8 and interferon-induced proteins, indicating that non-thrombotic immune-mediated injury plays a key role in OAPS pathogenesis (111).

Moreover, activated NK cells can inhibit spiral artery angiogenesis and vascular remodeling, exerting non-cytotoxic effects that impair embryo implantation and increase the risk of early pregnancy loss (112). Peripheral NKG2A^–^NKG2D^+^CD3^–^CD16^+^CD56^dim^ NK cell subsets are also elevated and positively correlate with antiphospholipid antibody levels, indicating that NK cell activation may be a key contributor to the immunopathology of OAPS (113).

#### 4.3.3 Monocyte and macrophage activation in pro-inflammatory responses

Previous studies have shown that monocytes from APS patients exhibit an activated phenotype, maintaining a pro-inflammatory state through upregulation of the NF-κB and MEK-1/ERK signaling pathways (114). Single-cell multi-omics analyses of the decidual microenvironment in OAPS patients have revealed broad immune cell imbalances. Notably, there is pronounced infiltration of CCR2^+^ monocyte-derived macrophages, which exert pro-inflammatory effects, thereby suppressing trophoblast proliferation and invasion and promoting apoptosis *in vitro* (115).

Moreover, IgG purified from OAPS patient serum can directly stimulate macrophages to produce CCL2 and TNF-α, contributing to trophoblast inflammatory injury. Mechanistically, antiphospholipid antibody/β2-glycoprotein I complex activate macrophages via the TLR4-NF-κB pathway, leading to upregulation of CCL2 and recruitment of CCR2^+^ monocyte-derived macrophages into the decidua. Mouse models further confirm that targeting TLR4 or CCR2 can mitigate antiphospholipid antibody-induced embryo resorption, highlighting the pivotal role of this pathway in OAPS pathogenesis (116).

## 5 Perspectives

### 5.1 Research perspectives

Future research should focus on elucidating the spatiotemporal dynamics of immune cell interactions at the maternal-fetal interface and on understanding how these immune networks evolve throughout gestation. Integrating multi-omics strategies will provide insights into the molecular connections that link complement activation, NET formation and cellular immune dysregulation, and the novel immune-mediated pathogenesis in maternal-fetal interface of OAPS. Identifying predictive biomarkers for distinguishing immune-mediated placental insufficiency from purely thrombotic pathology is also an important topic for early diagnosis and individualized risk assessment. Advanced placental organoid systems and humanized animal models will provide physiologically relevant platforms for mechanistic exploration and preclinical testing of novel immunomodulatory therapies.

### 5.2 Therapeutic perspectives

Emerging immunomodulatory strategies offer new opportunities to alleviate immune-mediated placental insufficiency in OAPS. Complement inhibitors, such as anti−C5 monoclonal antibodies and Factor B inhibitors, limit complement-driven inflammation and tissue damage (46, 117, 118). NET-targeting therapies, such as DNase I and PAD4 inhibitors, were reported to prevent NET formation and promote NET degradation, thereby reducing thrombosis and inflammatory amplification (79, 119, 120). B-cell modulation strategies focus on regulating B-cell survival and activation: Rituximab (anti-CD20) depletes autoreactive B cells, whereas Belimumab (BAFF inhibitor) suppresses B-cell survival, collectively reducing autoantibody production and supporting immune tolerance (121–124). Additionally, other targets, including the TLR4/NF−κB pathway, may mitigate cytokine release and innate immune activation, although evidence for these approaches remains largely theoretical.

Although most of these strategies are still at preclinical or early clinical stages, they collectively provide a mechanistic rationale for precision immunotherapy in patients refractory to conventional anticoagulation. Future clinical studies are warranted to rigorously evaluate their safety, efficacy, and optimal timing to ensure favorable maternal and fetal outcomes.

## 6 Conclusions

OAPS is a multifactorial autoimmune disorder in which aPLs disrupt pregnancy through both thrombotic and non-thrombotic mechanisms. Classical models have emphasized placental micro-thrombosis and the disruption of the Annexin V protective shield, but growing evidence highlights immune-mediated placental insufficiency as a central driver of disease pathogenesis. Complement activation, NET release, and dysregulated decidual NK cells, macrophages, and B cells collectively create a pro-inflammatory microenvironment at the maternal-fetal interface. This inflammatory milieu impairs trophoblast proliferation, invasion, and angiogenesis, ultimately compromising placental function. Furthermore, reciprocal amplification between NETs and the complement cascade fuels local inflammation and thrombogenicity, establishing a feed-forward loop of tissue injury.

Therapeutically, standard anticoagulation remains inadequate for a subset of patients, underscoring the need for immunomodulatory strategies that target complement, NETs, or autoreactive B cells. Collectively, these insights reframe OAPS as an immunopathological disorder beyond thrombosis and point toward precision therapeutics as the next frontier in improving pregnancy outcomes.

## References

[1] Knight JS Branch DW Ortel TL . Antiphospholipid syndrome: advances in diagnosis, pathogenesis, and management. BMJ. (2023) 380:e069717. doi: 10.1136/bmj-2021-069717, PMID: 36849186

[2] Ruiz-Irastorza G Crowther M Branch W Khamashta MA . Antiphospholipid syndrome. Lancet. (2010) 376:1498–509. doi: 10.1016/S0140-6736(10)60709-X, PMID: 20822807

[3] Schreiber K Sciascia S de Groot PG Devreese K Jacobsen S Ruiz-Irastorza G . Antiphospholipid syndrome. Nat Rev Dis Primers. (2018) 4:17104. doi: 10.1038/nrdp.2017.104, PMID: 29368699

[4] De Carolis S Tabacco S Rizzo F Giannini A Botta A Salvi S . Antiphospholipid syndrome: An update on risk factors for pregnancy outcome. Autoimmun Rev. (2018) 17:956–66. doi: 10.1016/j.autrev.2018.03.018, PMID: 30118899

[5] Ruiz-Irastorza G Egurbide M-V Ugalde J Aguirre C . High impact of antiphospholipid syndrome on irreversible organ damage and survival of patients with systemic lupus erythematosus. Arch Intern Med. (2004) 164:77–82. doi: 10.1001/archinte.164.1.77, PMID: 14718326

[6] Alijotas-Reig J Esteve-Valverde E Ferrer-Oliveras R Sáez-Comet L Lefkou E Mekinian A . The European Registry on Obstetric Antiphospholipid Syndrome (EUROAPS): A survey of 1000 consecutive cases. Autoimmun Rev. (2019) 18:406–14. doi: 10.1016/j.autrev.2018.12.006, PMID: 30772493

[7] Cervera R Piette J-C Font J Khamashta MA Shoenfeld Y Camps MT . Antiphospholipid syndrome: clinical and immunologic manifestations and patterns of disease expression in a cohort of 1,000 patients. Arthritis Rheum. (2002) 46:1019–27. doi: 10.1002/art.10187, PMID: 11953980

[8] Carrera-Marín A Romay-Penabad Z Papalardo E Reyes-Maldonado E García-Latorre E Vargas G . C6 knock-out mice are protected from thrombophilia mediated by antiphospholipid antibodies. Lupus. (2012) 21:1497–505. doi: 10.1177/0961203312458839, PMID: 22933620 PMC3923322

[9] Bouvier S Cochery-Nouvellon E Lavigne-Lissalde G Mercier E Marchetti T Balducchi J-P . Comparative incidence of pregnancy outcomes in treated obstetric antiphospholipid syndrome: the NOH-APS observational study. Blood. (2014) 123:404–13. doi: 10.1182/blood-2013-08-522623, PMID: 24200687

[10] Laskin CA Spitzer KA Clark CA Crowther MR Ginsberg JS Hawker GA . Low molecular weight heparin and aspirin for recurrent pregnancy loss: results from the randomized, controlled HepASA Trial. J Rheumatol. (2009) 36:279–87. doi: 10.3899/jrheum.080763, PMID: 19208560

[11] Alijotas-Reig J . Treatment of refractory obstetric antiphospholipid syndrome: the state of the art and new trends in the therapeutic management. Lupus. (2013) 22:6–17. doi: 10.1177/0961203312465782, PMID: 23151685

[12] Ca V Lw C . Histopathology in the placentae of women with antiphospholipid antibodies: A systematic review of the literature. Autoimmun Rev. (2015) 14:446–71. doi: 10.1016/j.autrev.2015.01.008, PMID: 25620498

[13] Dockery P Bermingham J Jenkins D . Structure-function relations in the human placenta. Biochem Soc Trans. (2000) 28:202–8. doi: 10.1042/bst0280202, PMID: 10816128

[14] Thliveris JA Baskett TF . Fine structure of the human placenta in prolonged pregnancy. Preliminary Rep Gynecol Obstet Invest. (1978) 9:40–8. doi: 10.1159/000300969, PMID: 710976

[15] Huppertz B Frank HG Kingdom JC Reister F Kaufmann P . Villous cytotrophoblast regulation of the syncytial apoptotic cascade in the human placenta. Histochem Cell Biol. (1998) 110:495–508. doi: 10.1007/s004180050311, PMID: 9826129

[16] Mayhew TM Leach L McGee R Ismail WW Myklebust R Lammiman MJ . Proliferation, differentiation and apoptosis in villous trophoblast at 13–41 weeks of gestation (including observations on annulate lamellae and nuclear pore complexes). Placenta. (1999) 20:407–22. doi: 10.1053/plac.1999.0399, PMID: 10419806

[17] Askelund KJ Chamley LW . Trophoblast deportation part I: review of the evidence demonstrating trophoblast shedding and deportation during human pregnancy. Placenta. (2011) 32:716–23. doi: 10.1016/j.placenta.2011.07.081, PMID: 21855136

[18] Pantham P Askelund KJ Chamley LW . Trophoblast deportation part II: a review of the maternal consequences of trophoblast deportation. Placenta. (2011) 32:724–31. doi: 10.1016/j.placenta.2011.06.019, PMID: 21839510

[19] Nakashima A Shima T Aoki A Kawaguchi M Yasuda I Tsuda S . Molecular and immunological developments in placentas. Hum Immunol. (2021) 82:317–24. doi: 10.1016/j.humimm.2021.01.012, PMID: 33581928 PMC8825288

[20] Fu Y-Y Ren C-E Qiao P-Y Meng Y-H . Uterine natural killer cells and recurrent spontaneous abortion. Am J Reprod Immunol. (2021) 86:e13433. doi: 10.1111/aji.13433, PMID: 33896061

[21] Levenson D Romero R Miller D Galaz J Garcia-Flores V Neshek B . The maternal-fetal interface at single-cell resolution: uncovering the cellular anatomy of the placenta and decidua. Am J Obstet Gynecol. (2025) 232:S55–79. doi: 10.1016/j.ajog.2024.12.032, PMID: 40253083 PMC13052991

[22] Sun F Wang S Du M . Functional regulation of decidual macrophages during pregnancy. J Reprod Immunol. (2021) 143:103264. doi: 10.1016/j.jri.2020.103264, PMID: 33360717

[23] Thum MY Bhaskaran S Abdalla HI Ford B Sumar N Shehata H . An increase in the absolute count of CD56dimCD16+CD69+ NK cells in the peripheral blood is associated with a poorer IVF treatment and pregnancy outcome. Hum Reprod. (2004) 19:2395–400. doi: 10.1093/humrep/deh378, PMID: 15319390

[24] Tong M Viall CA Chamley LW . Antiphospholipid antibodies and the placenta: a systematic review of their *in vitro* effects and modulation by treatment. Hum Reprod Update. (2015) 21:97–118. doi: 10.1093/humupd/dmu049, PMID: 25228006

[25] Abrahams VM Chamley LW Salmon JE . Antiphospholipid syndrome and pregnancy: pathogenesis to translation. Arthritis Rheumatol. (2017) 69:1710–21. doi: 10.1002/art.40136, PMID: 28445926 PMC5575987

[26] Alijotas-Reig J Esteve-Valverde E Anunciación-Llunell A Marques-Soares J Pardos-Gea J Miró-Mur F . Pathogenesis, diagnosis and management of obstetric antiphospholipid syndrome: A comprehensive review. J Clin Med. (2022) 11:675. doi: 10.3390/jcm11030675, PMID: 35160128 PMC8836886

[27] Campos B Chames M Lantry JM Bill JP Eis A Brockman D . Determination of non-bilayer phospholipid arrangements and their antibodies in placentae and sera of patients with hypertensive disorders of pregnancy. Placenta. (2006) 27:215–24. doi: 10.1016/j.placenta.2005.01.010, PMID: 16338467

[28] Katsuragawa H Rote NS Inoue T Narukawa S Kanzaki H Mori T . Monoclonal antiphosphatidylserine antibody reactivity against human first-trimester placental trophoblasts. Am J Obstet Gynecol. (1995) 172:1592–7. doi: 10.1016/0002-9378(95)90502-2, PMID: 7755077

[29] Lyden TW Vogt E Ng AK Johnson PM Rote NS . Monoclonal antiphospholipid antibody reactivity against human placental trophoblast. J Reprod Immunol. (1992) 22:1–14. doi: 10.1016/0165-0378(92)90002-l, PMID: 1522561

[30] Rand JH Wu X-X Quinn AS Chen PP McCrae KR Bovill EG . Human monoclonal antiphospholipid antibodies disrupt the annexin A5 anticoagulant crystal shield on phospholipid bilayers: evidence from atomic force microscopy and functional assay. Am J Pathol. (2003) 163:1193–200. doi: 10.1016/S0002-9440(10)63479-7, PMID: 12937161 PMC1868273

[31] Tait JF Gibson D Fujikawa K . Phospholipid binding properties of human placental anticoagulant protein-I, a member of the lipocortin family. J Biol Chem. (1989) 264:7944–9. doi: 10.1016/S0021-9258(18)83133-7, PMID: 2524476

[32] Rand JH Wu X-X Quinn AS Ashton AW Chen PP Hathcock JJ . Hydroxychloroquine protects the annexin A5 anticoagulant shield from disruption by antiphospholipid antibodies: evidence for a novel effect for an old antimalarial drug. Blood. (2010) 115:2292–9. doi: 10.1182/blood-2009-04-213520, PMID: 19965621 PMC2844013

[33] Rand JH Wu XX Guller S Scher J Andree HA Lockwood CJ . Antiphospholipid immunoglobulin G antibodies reduce annexin-V levels on syncytiotrophoblast apical membranes and in culture media of placental villi. Am J Obstet Gynecol. (1997) 177:918–23. doi: 10.1016/s0002-9378(97)70294-1, PMID: 9369845

[34] Rand JH Wu XX Andree HA Lockwood CJ Guller S Scher J . Pregnancy loss in the antiphospholipid-antibody syndrome–a possible thrombogenic mechanism. N Engl J Med. (1997) 337:154–60. doi: 10.1056/NEJM199707173370303, PMID: 9219701

[35] Rand JH Wu XX Guller S Gil J Guha A Scher J . Reduction of annexin-V (placental anticoagulant protein-I) on placental villi of women with antiphospholipid antibodies and recurrent spontaneous abortion. Am J Obstet Gynecol. (1994) 171:1566–72. doi: 10.1016/0002-9378(94)90403-0, PMID: 7802069

[36] Vogt E Ng AK Rote NS . Antiphosphatidylserine antibody removes annexin-V and facilitates the binding of prothrombin at the surface of a choriocarcinoma model of trophoblast differentiation. Am J Obstet Gynecol. (1997) 177:964–72. doi: 10.1016/s0002-9378(97)70302-8, PMID: 9369853

[37] Fox H . Pathology of the placenta. Major Probl Pathol. (1978) 7:343–8. 207935

[38] Kwak-Kim J Agcaoili MSL Aleta L Liao A Ota K Dambaeva S . Management of women with recurrent pregnancy losses and antiphospholipid antibody syndrome. Am J Reprod Immunol. (2013) 69:596–607. doi: 10.1111/aji.12114, PMID: 23521391

[39] Scambi C Ugolini S Tonello M Bortolami O De Franceschi L Castagna A . Complement activation in the plasma and placentas of women with different subsets of antiphospholipid syndrome. Am J Reprod Immunol. (2019) 82:e13185. doi: 10.1111/aji.13185, PMID: 31479579

[40] Cohen D Buurma A Goemaere NN Girardi G le Cessie S Scherjon S . Classical complement activation as a footprint for murine and human antiphospholipid antibody-induced fetal loss. J Pathol. (2011) 225:502–11. doi: 10.1002/path.2893, PMID: 21688269

[41] Shamonki JM Salmon JE Hyjek E Baergen RN . Excessive complement activation is associated with placental injury in patients with antiphospholipid antibodies. Am J Obstet Gynecol. (2007) 196:167.e1–5. doi: 10.1016/j.ajog.2006.10.879, PMID: 17306667 PMC2248299

[42] De Carolis S Botta A Santucci S Salvi S Moresi S Di Pasquo E . Complementemia and obstetric outcome in pregnancy with antiphospholipid syndrome. Lupus. (2012) 21:776–8. doi: 10.1177/0961203312444172, PMID: 22635230

[43] Reggia R Ziglioli T Andreoli L Bellisai F Iuliano A Gerosa M . Primary anti-phospholipid syndrome: any role for serum complement levels in predicting pregnancy complications? Rheumatol (Oxford). (2012) 51:2186–90. doi: 10.1093/rheumatology/kes225, PMID: 22923750

[44] Girardi G Berman J Redecha P Spruce L Thurman JM Kraus D . Complement C5a receptors and neutrophils mediate fetal injury in the antiphospholipid syndrome. J Clin Invest. (2003) 112:1644–54. doi: 10.1172/JCI18817, PMID: 14660741 PMC281643

[45] Salmon JE Girardi G . The role of complement in the antiphospholipid syndrome. Curr Dir Autoimmun. (2004) 7:133–48. doi: 10.1159/000075690, PMID: 14719378

[46] Thurman JM Kraus DM Girardi G Hourcade D Kang HJ Royer PA . A novel inhibitor of the alternative complement pathway prevents antiphospholipid antibody-induced pregnancy loss in mice. Mol Immunol. (2005) 42:87–97. doi: 10.1016/j.molimm.2004.07.043, PMID: 15488947

[47] Tincani A Cavazzana I Ziglioli T Lojacono A De Angelis V Meroni P . Complement activation and pregnancy failure. Clin Rev Allergy Immunol. (2010) 39:153–9. doi: 10.1007/s12016-009-8183-5, PMID: 19936969

[48] Porter RR Reid KB . The biochemistry of complement. Nature. (1978) 275:699–704. doi: 10.1038/275699a0, PMID: 703835

[49] Chaturvedi S Brodsky RA McCrae KR . Complement in the pathophysiology of the antiphospholipid syndrome. Front Immunol. (2019) 10:449. doi: 10.3389/fimmu.2019.00449, PMID: 30923524 PMC6426753

[50] Tedesco F Borghi MO Gerosa M Chighizola CB Macor P Lonati PA . Pathogenic role of complement in antiphospholipid syndrome and therapeutic implications. Front Immunol. (2018) 9:1388. doi: 10.3389/fimmu.2018.01388, PMID: 29971066 PMC6018396

[51] Pangburn MK . Initiation of the alternative pathway of complement and the history of “tickover. Immunol Rev. (2023) 313:64–70. doi: 10.1111/imr.13130, PMID: 36089768

[52] Breen KA Seed P Parmar K Moore GW Stuart-Smith SE Hunt BJ . Complement activation in patients with isolated antiphospholipid antibodies or primary antiphospholipid syndrome. Thromb Haemost. (2012) 107:423–9. doi: 10.1160/TH11-08-0554, PMID: 22234447

[53] Holers VM Girardi G Mo L Guthridge JM Molina H Pierangeli SS . Complement C3 activation is required for antiphospholipid antibody-induced fetal loss. J Exp Med. (2002) 195:211–20. doi: 10.1084/jem.200116116, PMID: 11805148 PMC2193604

[54] Kaneko K Ozawa N Murashima A . Obstetric anti-phospholipid syndrome: from pathogenesis to treatment. Immunol Med. (2022) 45:79–93. doi: 10.1080/25785826.2021.1969116, PMID: 34470570

[55] Ricklin D Mastellos DC Reis ES Lambris JD . The renaissance of complement therapeutics. Nat Rev Nephrol. (2018) 14:26–47. doi: 10.1038/nrneph.2017.156, PMID: 29199277 PMC5805379

[56] Spiller OB Criado-García O Rodríguez De Córdoba S Morgan BP . Cytokine-mediated up-regulation of CD55 and CD59 protects human hepatoma cells from complement attack. Clin Exp Immunol. (2000) 121:234–41. doi: 10.1046/j.1365-2249.2000.01305.x, PMID: 10931136 PMC1905706

[57] Barilla-LaBarca ML Liszewski MK Lambris JD Hourcade D Atkinson JP . Role of membrane cofactor protein (CD46) in regulation of C4b and C3b deposited on cells. J Immunol. (2002) 168:6298–304. doi: 10.4049/jimmunol.168.12.6298, PMID: 12055245

[58] Cardone J Le Friec G Kemper C . CD46 in innate and adaptive immunity: an update. Clin Exp Immunol. (2011) 164:301–11. doi: 10.1111/j.1365-2249.2011.04400.x, PMID: 21488871 PMC3087925

[59] Harris CL Pettigrew DM Lea SM Morgan BP . Decay-accelerating factor must bind both components of the complement alternative pathway C3 convertase to mediate efficient decay. J Immunol. (2007) 178:352–9. doi: 10.4049/jimmunol.178.1.352, PMID: 17182573

[60] Kuttner-Kondo L Hourcade DE Anderson VE Muqim N Mitchell L Soares DC . Structure-based mapping of DAF active site residues that accelerate the decay of C3 convertases. J Biol Chem. (2007) 282:18552–62. doi: 10.1074/jbc.M611650200, PMID: 17395591

[61] Zhang Y Jin S . Mitigating placental injuries through up-regulating DAF in experimental APS mice: new mechanism of progesterone. Clin Exp Immunol. (2019) 197:376–86. doi: 10.1111/cei.13313, PMID: 31091357 PMC6693963

[62] Wirstlein PK Jasiński P Rajewski M Goździewicz T Skrzypczak J . Complement inhibitory proteins expression in placentas of thrombophilic women. Folia Histochem Cytobiol. (2012) 50:460–7. doi: 10.5603/19758, PMID: 23042280

[63] Jorch SK Kubes P . An emerging role for neutrophil extracellular traps in noninfectious disease. Nat Med. (2017) 23:279–87. doi: 10.1038/nm.4294, PMID: 28267716

[64] Carmona-Rivera C Zhao W Yalavarthi S Kaplan MJ . Neutrophil extracellular traps induce endothelial dysfunction in systemic lupus erythematosus through the activation of matrix metalloproteinase-2. Ann Rheum Dis. (2015) 74:1417–24. doi: 10.1136/annrheumdis-2013-204837, PMID: 24570026 PMC4143484

[65] Hakkim A Fürnrohr BG Amann K Laube B Abed UA Brinkmann V . Impairment of neutrophil extracellular trap degradation is associated with lupus nephritis. Proc Natl Acad Sci U.S.A. (2010) 107:9813–8. doi: 10.1073/pnas.0909927107, PMID: 20439745 PMC2906830

[66] Lu Y Dong Y Zhang Y Shen D Wang X Ge R . Antiphospholipid antibody-activated NETs exacerbate trophoblast and endothelial cell injury in obstetric antiphospholipid syndrome. J Cell Mol Med. (2020) 24:6690–703. doi: 10.1111/jcmm.15321, PMID: 32369873 PMC7299718

[67] Yalavarthi S Gould TJ Rao AN Mazza LF Morris AE Núñez-Álvarez C . Release of neutrophil extracellular traps by neutrophils stimulated with antiphospholipid antibodies: a newly identified mechanism of thrombosis in the antiphospholipid syndrome. Arthritis Rheumatol. (2015) 67:2990–3003. doi: 10.1002/art.39247, PMID: 26097119 PMC4626310

[68] Nayak L Sweet DR Thomas A Lapping SD Kalikasingh K Madera A . A targetable pathway in neutrophils mitigates both arterial and venous thrombosis. Sci Transl Med. (2022) 14:eabj7465. doi: 10.1126/scitranslmed.abj7465, PMID: 36044595 PMC10318551

[69] Petretto A Bruschi M Pratesi F Croia C Candiano G Ghiggeri G . Neutrophil extracellular traps (NET) induced by different stimuli: A comparative proteomic analysis. PloS One. (2019) 14:e0218946. doi: 10.1371/journal.pone.0218946, PMID: 31283757 PMC6613696

[70] De Bont CM Boelens WC Pruijn GJM . NETosis, complement, and coagulation: a triangular relationship. Cell Mol Immunol. (2019) 16:19–27. doi: 10.1038/s41423-018-0024-0, PMID: 29572545 PMC6318284

[71] Leffler J Stojanovich L Shoenfeld Y Bogdanovic G Hesselstrand R Blom AM . Degradation of neutrophil extracellular traps is decreased in patients with antiphospholipid syndrome. Clin Exp Rheumatol. (2014) 32:66–70., PMID: 24295292

[72] Zuo Y Yalavarthi S Gockman K Madison JA Gudjonsson JE Kahlenberg JM . Anti–neutrophil extracellular trap antibodies and impaired neutrophil extracellular trap degradation in antiphospholipid syndrome. Arthritis Rheumatol. (2020) 72(12):2130–35. doi: 10.1002/art.41460, PMID: 32729667 PMC7722115

[73] Wigerblad G Kaplan MJ . Neutrophil extracellular traps in systemic autoimmune and autoinflammatory diseases. Nat Rev Immunol. (2023) 23:274–88. doi: 10.1038/s41577-022-00787-0, PMID: 36257987 PMC9579530

[74] Zhou Y Xu Z Liu Z . Impact of neutrophil extracellular traps on thrombosis formation: new findings and future perspective. Front Cell Infect Microbiol. (2022) 12:910908. doi: 10.3389/fcimb.2022.910908, PMID: 35711663 PMC9195303

[75] Saffarzadeh M Juenemann C Queisser MA Lochnit G Barreto G Galuska SP . Neutrophil extracellular traps directly induce epithelial and endothelial cell death: a predominant role of histones. PloS One. (2012) 7:e32366. doi: 10.1371/journal.pone.0032366, PMID: 22389696 PMC3289648

[76] Noubouossie DF Whelihan MF Yu Y-B Sparkenbaugh E Pawlinski R Monroe DM . *In vitro* activation of coagulation by human neutrophil DNA and histone proteins but not neutrophil extracellular traps. Blood. (2017) 129:1021–9. doi: 10.1182/blood-2016-06-722298, PMID: 27919911 PMC5324715

[77] Wang H Kim SJ Lei Y Wang S Wang H Huang H . Neutrophil extracellular traps in homeostasis and disease. Sig Transduct Target Ther. (2024) 9:235. doi: 10.1038/s41392-024-01933-x, PMID: 39300084 PMC11415080

[78] Yang X Li L Liu J Lv B Chen F . Extracellular histones induce tissue factor expression in vascular endothelial cells via TLR and activation of NF-κB and AP-1. Thromb Res. (2016) 137:211–8. doi: 10.1016/j.thromres.2015.10.012, PMID: 26476743

[79] Butt A Sharda A Lee AI Knight JS . Analytical review: neutrophil extracellular traps and antiphospholipid syndrome. Transfus Med Rev. (2025), 150909. doi: 10.1016/j.tmrv.2025.150909, PMID: 40783311

[80] Foret T Dufrost V Salomon du Mont L Costa P Lakomy C Lagrange J . A new pro-thrombotic mechanism of neutrophil extracellular traps in antiphospholipid syndrome: impact on activated protein C resistance. Rheumatol (Oxford). (2022) 61:2993–8. doi: 10.1093/rheumatology/keab853, PMID: 34791113

[81] Mineo C Shaul PW Bermas BL . The pathogenesis of obstetric APS: a 2023 update. Clin Immunol. (2023) 255:109745. doi: 10.1016/j.clim.2023.109745, PMID: 37625670 PMC11366079

[82] Wang C Li A Zhang C Yang Z Yang X Qi W . Neutrophil extracellular traps aggravate placental injury in OAPS by facilitating activation of BNIP3 mediated mitophagy. Free Radic Biol Med. (2025) 235:109–23. doi: 10.1016/j.freeradbiomed.2025.04.038, PMID: 40286883

[83] Maki MS Ali MS Rawi HZ . The association of matrix metalloproteinase-9 and fetal fibronectin in the first trimester threatened miscarriage. Immunopathol Persa. (2024) 10:e40644–4. doi: 10.34172/ipp.2024.40644

[84] Leffler J Martin M Gullstrand B Tydén H Lood C Truedsson L . Neutrophil extracellular traps that are not degraded in systemic lupus erythematosus activate complement exacerbating the disease. J Immunol. (2012) 188:3522–31. doi: 10.4049/jimmunol.1102404, PMID: 22345666

[85] Wang H Wang C Zhao M-H Chen M . Neutrophil extracellular traps can activate alternative complement pathways. Clin Exp Immunol. (2015) 181:518–27. doi: 10.1111/cei.12654, PMID: 25963026 PMC4557387

[86] Yuen J Pluthero FG Douda DN Riedl M Cherry A Ulanova M . NETosing neutrophils activate complement both on their own NETs and bacteria via alternative and non-alternative pathways. Front Immunol. (2016) 7:137. doi: 10.3389/fimmu.2016.00137, PMID: 27148258 PMC4831636

[87] O’Flynn J Dixon KO Faber Krol MC Daha MR van Kooten C . Myeloperoxidase directs properdin-mediated complement activation. J Innate Immun. (2014) 6:417–25. doi: 10.1159/000356980, PMID: 24355864 PMC6741500

[88] Schneider AE Sándor N Kárpáti É Józsi M . Complement factor H modulates the activation of human neutrophil granulocytes and the generation of neutrophil extracellular traps. Mol Immunol. (2016) 72:37–48. doi: 10.1016/j.molimm.2016.02.011, PMID: 26938503

[89] Chen Y Li X Lin X Liang H Liu X Zhang X . Complement C5a induces the generation of neutrophil extracellular traps by inhibiting mitochondrial STAT3 to promote the development of arterial thrombosis. Thromb J. (2022) 20:24. doi: 10.1186/s12959-022-00384-0, PMID: 35488279 PMC9051782

[90] Huang Y-M Wang H Wang C Chen M Zhao M-H . Promotion of hypercoagulability in antineutrophil cytoplasmic antibody-associated vasculitis by C5a-induced tissue factor-expressing microparticles and neutrophil extracellular traps. Arthritis Rheumatol. (2015) 67:2780–90. doi: 10.1002/art.39239, PMID: 26097236

[91] Grossi C Capitani N Benagiano M Baldari CT Della Bella C Macor P . Beta 2 glycoprotein I and neutrophil extracellular traps: Potential bridge between innate and adaptive immunity in anti-phospholipid syndrome. Front Immunol. (2023) 13:1076167. doi: 10.3389/fimmu.2022.1076167, PMID: 36700193 PMC9868732

[92] Liu J-C Zeng Q Duan Y-G Yeung WSB Li RHW Ng EHY . B cells: roles in physiology and pathology of pregnancy. Front Immunol. (2024) 15:1456171. doi: 10.3389/fimmu.2024.1456171, PMID: 39434884 PMC11491347

[93] Hisada R Kato M Sugawara E Kanda M Fujieda Y Oku K . Circulating plasmablasts contribute to antiphospholipid antibody production, associated with type I interferon upregulation. J Thromb Haemost. (2019) 17:1134–43. doi: 10.1111/jth.14427, PMID: 30864219

[94] Li X-Y Duan H-J Liu X-Y Deng X-L . Change of serum B-cell activating factor level in patients with positive antiphospholipid antibodies and previous adverse pregnancy outcomes and its significance. Chin Med J (Engl). (2020) 133:2287–94. doi: 10.1097/CM9.0000000000000948, PMID: 32842014 PMC7546878

[95] López P Rodríguez-Carrio J Caminal-Montero L Mozo L Suárez A . A pathogenic IFNα, BLyS and IL-17 axis in Systemic Lupus Erythematosus patients. Sci Rep. (2016) 6:20651. doi: 10.1038/srep20651, PMID: 26847824 PMC4742957

[96] Thompson N Isenberg DA Jury EC Ciurtin C . Exploring BAFF: its expression, receptors and contribution to the immunopathogenesis of Sjögren’s syndrome. Rheumatol (Oxford). (2016) 55:1548–55. doi: 10.1093/rheumatology/kev420, PMID: 26790457

[97] Chang SK Arendt BK Darce JR Wu X Jelinek DF . A role for BLyS in the activation of innate immune cells. Blood. (2006) 108:2687–94. doi: 10.1182/blood-2005-12-017319, PMID: 16825497 PMC1895592

[98] Velásquez M Rojas M Abrahams VM Escudero C Cadavid ÁP . Mechanisms of endothelial dysfunction in antiphospholipid syndrome: association with clinical manifestations. Front Physiol. (2018) 9:1840. doi: 10.3389/fphys.2018.01840, PMID: 30627104 PMC6309735

[99] Nutt SL Hodgkin PD Tarlinton DM Corcoran LM . The generation of antibody-secreting plasma cells. Nat Rev Immunol. (2015) 15:160–71. doi: 10.1038/nri3795, PMID: 25698678

[100] Zhou Y Zhang Y Han J Yang M Zhu J Jin T . Transitional B cells involved in autoimmunity and their impact on neuroimmunological diseases. J Transl Med. (2020) 18:131. doi: 10.1186/s12967-020-02289-w, PMID: 32183811 PMC7079408

[101] Zhang Y Lin M Hao X Ping M Zhang H Zheng J . Imbalance of circulating CTLA4+ follicular helper and follicular regulatory T cells in obstetric antiphospholipid syndrome. Clin Exp Med. (2022) 22:27–36. doi: 10.1007/s10238-021-00720-0, PMID: 34002285

[102] Tanimura K Jin H Suenaga T Morikami S Arase N Kishida K . β2-Glycoprotein I/HLA class II complexes are novel autoantigens in antiphospholipid syndrome. Blood. (2015) 125:2835–44. doi: 10.1182/blood-2014-08-593624, PMID: 25733579 PMC4424631

[103] Liu T Han J Zhang R Tang Z Yi G Gong W . Characteristics of purified anti-β2GPI IgG N-glycosylation associate with thrombotic, obstetric and catastrophic antiphospholipid syndrome. Rheumatol (Oxford). (2022) 61:1243–54. doi: 10.1093/rheumatology/keab416, PMID: 34015111

[104] Wang Y Li S Meng J Yu R Wang Q Tian X . Changes in serum immunoglobulin G glycosylation patterns for antiphospholipid syndrome patients with lectin microarray. Scand J Immunol. (2024) 99:e13366. doi: 10.1111/sji.13366, PMID: 38720518

[105] Cartwright JE James-Allan L Buckley RJ Wallace AE . The role of decidual NK cells in pregnancies with impaired vascular remodelling. J Reprod Immunol. (2017) 119:81–4. doi: 10.1016/j.jri.2016.09.002, PMID: 27680579

[106] Chen P Zhou L Chen J Lu Y Cao C Lv S . The immune atlas of human deciduas with unexplained recurrent pregnancy loss. Front Immunol. (2021) 12:689019. doi: 10.3389/fimmu.2021.689019, PMID: 34168655 PMC8218877

[107] Guo C Cai P Jin L Sha Q Yu Q Zhang W . Single-cell profiling of the human decidual immune microenvironment in patients with recurrent pregnancy loss. Cell Discov. (2021) 7:1. doi: 10.1038/s41421-020-00236-z, PMID: 33390590 PMC7779601

[108] Pan D Liu Q Du L Yang Y Jiang G . Polarization disorder of decidual NK cells in unexplained recurrent spontaneous abortion revealed by single-cell transcriptome analysis. Reprod Biol Endocrinol. (2022) 20:108. doi: 10.1186/s12958-022-00980-9, PMID: 35897028 PMC9327377

[109] Wang F Jia W Fan M Shao X Li Z Liu Y . Single-cell immune landscape of human recurrent miscarriage. Genomics Proteomics Bioinf. (2021) 19:208–22. doi: 10.1016/j.gpb.2020.11.002, PMID: 33482359 PMC8602400

[110] Clark DA . Popular myths in reproductive immunology. J Reprod Immunol. (2014) 104–105:54–62. doi: 10.1016/j.jri.2014.06.002, PMID: 25087657

[111] Liu Y Gao S Zhao Y Wang H Pan Q Shao Q . Decidual natural killer cells: A good nanny at the maternal-fetal interface during early pregnancy. Front Immunol. (2021) 12:663660. doi: 10.3389/fimmu.2021.663660, PMID: 34054831 PMC8149889

[112] Manukyan G Kriegova E Slavik L Mikulkova Z Ulehlova J Martirosyan A . Antiphospholipid antibody-mediated NK cell cytotoxicity. J Reprod Immunol. (2023) 155:103791. doi: 10.1016/j.jri.2022.103791, PMID: 36621092

[113] Zhang Y Zhao Y Si W Yang B Lin M Zheng J . Increased peripheral NKG2A-NKG2D+CD3-CD16+CD56dim NK cell subset was positively correlated with antiphospholipid antibodies in patients of obstetric antiphospholipid syndrome. Immunol Invest. (2022) 51:425–37. doi: 10.1080/08820139.2020.1835949, PMID: 33103514

[114] Sorice M Longo A Capozzi A Garofalo T Misasi R Alessandri C . Anti-beta2-glycoprotein I antibodies induce monocyte release of tumor necrosis factor alpha and tissue factor by signal transduction pathways involving lipid rafts. Arthritis Rheum. (2007) 56:2687–97. doi: 10.1002/art.22802, PMID: 17665396

[115] Lu C Gao R Qing P Zeng X Liao X Cheng M . Single-cell transcriptome analyses reveal disturbed decidual homoeostasis in obstetric antiphospholipid syndrome. Ann Rheum Dis. (2024) 83:624–37. doi: 10.1136/ard-2023-224930, PMID: 38331588

[116] Gao R Qing P Chen H Hu Z Yang Q Lu C . Deciphering the role and mechanism of decidual monocyte-derived macrophage infiltration in obstetric antiphospholipid syndrome at single-cell resolution. Adv Sci (Weinh). (2025) 12:e03480. doi: 10.1002/advs.202503480, PMID: 40776470 PMC12591211

[117] Shapira I Andrade D Allen SL Salmon JE . Brief report: induction of sustained remission in recurrent catastrophic antiphospholipid syndrome via inhibition of terminal complement with eculizumab. Arthritis Rheum. (2012) 64:2719–23. doi: 10.1002/art.34440, PMID: 22354668

[118] Erkan D Salmon JE . The role of complement inhibition in thrombotic angiopathies and antiphospholipid syndrome. Turk J Haematol. (2016) 33:1–7. doi: 10.4274/tjh.2015.0197, PMID: 27020721 PMC4805354

[119] Barnado A Crofford LJ Oates JC . At the Bedside: Neutrophil extracellular traps (NETs) as targets for biomarkers and therapies in autoimmune diseases. J Leukoc Biol. (2016) 99:265–78. doi: 10.1189/jlb.5BT0615-234R, PMID: 26658004 PMC6608010

[120] Giaglis S Hahn S Hasler P . The NET outcome”: are neutrophil extracellular traps of any relevance to the pathophysiology of autoimmune disorders in childhood? Front Pediatr. (2016) 4:97. doi: 10.3389/fped.2016.00097, PMID: 27679792 PMC5020135

[121] Yazici A Yazirli B Erkan D . Belimumab in primary antiphospholipid syndrome. Lupus. (2017) 26:1123–4. doi: 10.1177/0961203316682102, PMID: 27913749

[122] Klemm P Müller-Ladner U Tarner IH Lange U Hudowenz O . Belimumab reduces antiphospholipid antibodies in primary triple-positive antiphospholipid syndrome. Autoimmun Rev. (2020) 19:102594. doi: 10.1016/j.autrev.2020.102594, PMID: 32535091

[123] Berman H Rodríguez-Pintó I Cervera R Morel N Costedoat-Chalumeau N Erkan D . Catastrophic Antiphospholipid Syndrome (CAPS) Registry Project Group (European Forum on Antiphospholipid Antibodies). Rituximab use in the catastrophic antiphospholipid syndrome: descriptive analysis of the CAPS registry patients receiving rituximab. Autoimmun Rev. (2013) 12:1085–90. doi: 10.1016/j.autrev.2013.05.004, PMID: 23777822

[124] Agmon-Levin N Berman M Harel L Lidar M Drori T Hajyahia S . Rituximab for refractory manifestations of the antiphospholipid syndrome: a multicentre Israeli experience. Clin Exp Rheumatol. (2021) 39:1049–55. doi: 10.55563/clinexprheumatol/cc5taf, PMID: 33124581

